# O.4.6-2 Evaluation of a school-based physical activity and sport programs policy: the importance of teacher training

**DOI:** 10.1093/eurpub/ckad133.221

**Published:** 2023-09-11

**Authors:** Alexandra Valencia-Peris, Isabel Pérez-Herráez, Carlos Evangelio, Roberto Ferriz-Morell, Jorge Lizandra

**Affiliations:** University of Valencia, Spain; University of Valencia, Spain; University of Valencia, Spain; University of Castilla-La Mancha, Spain; alexandra.valencia@uv.es; University of Valencia, Spain; University of Valencia, Spain

## Abstract

**Purpose:**

School-based interventions are considered the best approach to increase young people’s physical activity levels, although their effectiveness and sustainability remain unclear. This work aims to determine if a school-based physical activity and sports programs (PASP) policy is being evaluated by schools.

**Methods:**

A representative sample of 257 Active schools in the Valencian Community (Spain) participated in the study. The coordinator of each school answered an ad-hoc on-line questionnaire, consisting of 47 open and closed questions aimed to identify the Active school coordinators’ profiles and the actions implemented to foster PASP and their assessment. Descriptive statistics and chi-square tests were conducted on SPSS Statistics v. 26.0 software.

**Results:**

Fifty-one percent of the Active schools had a commission in charge of designing, implementing, and evaluating the PASP (30.7% formed by teachers, 1.6% by teachers and students and 4.3% by teachers, students, and their families). However, in many cases this commission never actually met (49%) and the rest met with the following frequency: once a school year (14.4%), once a term (4.3%), once a month (1.6%) and weekly (30.7%). Nearly 80% of the schools carried out some form of PASP evaluation: by diagnosis (19.5%), monitoring (43.2%), effectiveness (44.7%) or final meetings in the educational cycle (20.6%). Both having a commission *χ*^*2*^_*(1)*_=9.385; *p*<.05) and evaluating the PASP *χ*^*2*^_*(1)*_=14.154; *p*<.05) depended on whether the school coordinators had received specific training related to promoting physical activity, sports and health.

**Conclusions:**

Only half of the Active schools had PASP evaluation commissions and most of these met very infrequently. Although most of the Active schools did evaluate their interventions, less than half focused on their effectiveness. Teacher training was a determining factor in both creating these commissions and developing PASP assessment processes. These results show the importance of improving pre- and in-service teacher training, especially for Physical Education teachers in relation to health education and PASP promotion in school settings.

**Funding:**

This study was funded by Grant AICO/2021/342 of the CIUCSD (Spain), Postdoc grant Margarita Salas funded by the University of Castilla-La Mancha [MS2021] and predoctoral contract UV-INV-PREDOC20-1339837 funded by University of Valencia.

